# Il faut démanteler le statu quo et promouvoir des politiques pour la santé, le bien-être et l’équité : un prélude à l’ « IUHPE2022 »

**DOI:** 10.1177/17579759211044290

**Published:** 2021-09-17

**Authors:** Brittany Wenniserí:iostha Jock, Carole Clavier, Evelyne de Leeuw, Katherine L. Frohlich

**Affiliations:** 1 Centre for Indigenous Peoples’ Nutrition & Environment (CINE), McGill University, Montréal, Québec, Canada; 2 Département de Science Politique, Université du Québec à Montréal, Montréal, Québec, Canada; 3 Centre for Health Equity Training, Research & Evaluation (CHETRE) (Centre de Formation, de Recherche et d’Evaluation sur l’Equité en Santé) UNSW Australia Research Centre for Primary Health Care & Equity, A Unit of Population Health; membre de l’Institut Ingham, Sydney, Australie; 4 École de Santé Publique (ESPUM) et Centre de Recherche en Santé Publique (CReSP), Université de Montréal, Montréal, Canada.

**Keywords:** plaidoyer (y compris plaidoyer médiatique), collaboration/partenariats, déterminants de la santé, autonomisation/pouvoir, équité/justice sociale, santé mondiale/mondialisation, promotion de la santé

## Abstract

Le prochain rassemblement international de la famille mondiale de la promotion de la santé aura lieu à Montréal en mai 2022. La 24^ème^ Conférence de l’UIPES a pour thème « Promouvoir des politiques pour la santé, le bien-être et l’équité ». Les organisateurs de la Conférence ont décidé de transcender la rhétorique des « suspects habituels » et de construire un programme de conférence qui remette vraiment en question les notions clés qui sous-tendent la promotion de la santé. Dans ce commentaire, les membres des Comités scientifiques mondial et canadien réfléchissent à l’état des lieux et aux possibilités à venir. Nous proposons les trois thèmes suivants : (a) saisir les opportunités dans les changements actuels en repérant les bouleversements ou les moments charnières qui représentent une promesse ou des fenêtres d’opportunités, qu’il s’agisse de défis à relever comme les pandémies, de changements climatiques ou géopolitiques, de troubles sociaux ou de l’apport de l’évolution des technologies ; (b) s’affranchir des visions conventionnelles du monde qui ne privilégient que les solutions commerciales, aller vers la décolonisation des pratiques et des systèmes pour s’émanciper des conceptions basées sur les divisions entre le Nord et le Sud ; et (c) innover en brisant les silos entre les disciplines, les frontières et les identités enracinées dans nos pratiques et notre compréhension de l’innovation.

La promotion de la santé en tant que champ de pratique et de recherche continue d’évoluer. En tant que mouvement mondial, la communauté de la promotion de la santé fait entendre de nombreuses voix. Il est toutefois difficile de continuer à trouver un équilibre entre des techniques et des approches élégantes et sophistiquées, d’une part, et des problèmes de santé bien concrets et souvent aigus, d’autre part.

Les conférences mondiales de l’Union internationale de promotion de la santé et d’éducation pour la santé ont – la plupart du temps – réussi à trouver ce juste équilibre. Le prochain rassemblement international de la famille de la promotion de la santé mondiale se tiendra à Montréal, en mai 2022. La 24e conférence de l’UIPES a pour thème « Promouvoir des politiques pour la santé, le bien-être et l’équité ». Les organisateurs de la conférence ont décidé de transcender la rhétorique des « suspects habituels » qui consiste à viser l’équité en santé (par des discussions par exemple sur des thématiques comme les déterminants sociaux de la santé et la santé dans toutes les politiques) pour plutôt examiner les causes profondes des iniquités en santé et leurs déterminants structurels, y compris ceux d’ordre politique, économique, environnemental, culturel et social. Les Comités scientifiques de l’« IUHPE 2022 » construisent un programme de conférence qui remet vraiment en question les fondements et les orientations des politiques en matière de santé, de bien-être et d’équité pour la promotion de la santé. Dans ce commentaire, les membres des Comités scientifiques mondial et canadien réfléchissent à l’état des lieux et aux possibilités à venir.

La promotion de la santé reste le processus qui permet aux individus, aux groupes et aux communautés d’avoir davantage de maîtrise des déterminants de leur santé et davantage de moyens de les améliorer. Pour parvenir à un état de complet bien-être physique, mental et social, l’individu, ou le groupe, doit pouvoir identifier et réaliser ses ambitions, satisfaire ses besoins et évoluer avec son milieu ou s’y adapter (1). Notre communauté se positionne comme un mouvement social positif, qui s’engage avec des vues optimistes dans ce qui détermine la santé et le bien-être humain, écologique et planétaire. Nous luttons également pour la justice sociale et la réduction de toutes les inégalités (sociales, écologiques, culturelles, ou créées par tout autre paramètre), qui influencent négativement la santé.

Cependant, au moment où nous écrivons ce commentaire, la maladie à coronavirus 2019 (COVID-19), fait des ravages dans les populations et les économies, révélant non seulement notre fragilité face aux nouvelles maladies infectieuses, mais aussi combien les changements climatiques, les inégalités sociales et raciales systémiques, et les failles de nos systèmes politiques et économiques pèsent sur notre bien-être collectif. Il ne s’agit pas seulement d’une épidémie de dimension mondiale (une « pandémie »), mais bien d’une « syndémie » – une coalescence systémique d’événements sanitaires et sociaux qui expose des lignes de faille critiques à travers le monde (2). Horton (3), le rédacteur en chef de *The Lancet*, s’est écarté, à juste titre, de la notion de « syndémie » proposée à l’origine par Singer, plus axée sur l’épidémiologie. La tragédie de la syndémie de COVID-19 n’est pas seulement l’impact inéquitable d’une série de comorbidités (cliniques) désastreuses, mais c’est aussi l’effet d’un monde qui permet la mort de centaines de milliers de personnes dans des pays superficiellement riches et puissants. La syndémie est aussi le résultat d’un mépris pervers pour de larges pans de populations défavorisées qui maintiennent précairement des économies néolibérales à flot. Elle souligne, par exemple, que nous sous-valorisons et sous-payons des millions de travailleurs essentiels. Nous sommes témoins du prix élevé que nous payons – sur les plans environnemental, social et de la santé – pour nos économies compétitives sous forte pression.

Des événements comme le meurtre de George Floyd aux États-Unis, le mouvement « Black Lives Matter » et les inégalités effroyables de la pandémie qui se sont avérées dans de nombreuses communautés racialisées et défavorisées sur le plan économique et social nous amènent à marquer une pause pour réfléchir aux insuffisances de nos approches politiques. Qu’elles soient apocryphes, optimistes ou cyniques, les données empiriques dans le domaine de la recherche politique sont claires : les urgences et les catastrophes inspirent le changement. Cette notion de moments importants et limités de bouleversement et de changement potentiel émerge des travaux de John Kingdon sur la théorie des courants multiples (4) et de la pensée dite des « équilibres ponctués » (5). Nous aspirons à une promotion de la santé qui repousse les limites des politiques favorables à la santé, au bien-être et à l’équité. Nous voulons réfléchir à la manière dont peuvent être repensées l’intégration des services de l’Etat et l’intégration de la santé dans toutes les politiques pour remédier efficacement aux inégalités que la promotion de la santé cherche à vaincre. Avec la syndémie et les 70 ans d’histoire du champ que nous appelons promotion de la santé, vient la question : quels sont les contours et les grands enjeux de la promotion de la santé à laquelle nous aspirons pour les générations futures ?

Les organisateurs de l’ « IUHPE2022 » ont identifié trois thèmes pour nous aider à réfléchir à la situation actuelle de la promotion de la santé; ce que nous faisons bien et ce à quoi nous pouvons aspirer pour mieux comprendre, nous engager et changer.

Nous avons formulé ces thèmes comme suit :

- Saisir les opportunités dans les changements actuels en repérant les bouleversements ou les moments charnières qui représentent une promesse ou des fenêtres d’opportunités, qu’il s’agisse de défis à relever comme les pandémies, de changements climatiques ou géopolitiques, de troubles sociaux ou de l’apport de l’évolution des technologies ;
- S’affranchir des visions conventionnelles du monde qui ne privilégient que les solutions commerciales, aller vers la décolonisation des pratiques et des systèmes pour s’émanciper des conceptions basées sur les divisions entre le Nord et le Sud ; et
- Innover en brisant les silos entre les disciplines, les frontières et les identités enracinées dans nos pratiques et notre compréhension de l’innovation.

Les conversations que nous souhaitons avoir à notre conférence devraient être intéressantes et animées, parfois même artistiques et pleines d’humour. Elles devraient être inspirantes, mais aussi difficiles. Nous reconnaissons que les dizaines de milliers de membres – individuels et institutionnels, détenteurs ou non d’une carte – qui composent la communauté mondiale de la promotion de la santé forment un lot d’une grande diversité. Ce qui peut être difficile pour certains peut être réconfortant pour d’autres. Ce qui est une pratique courante dans le Sud peut se révéler une innovation radicale dans le Nord. Ce commentaire vise à établir des points communs pour nous tous.

## Saisir les opportunités engendrées par un bouleversement mondial

Alors que le terme « innovation disruptive » découle d’une théorie utilisée dans le monde des affaires (6), fermement ancrée dans les économies néolibérales que la promotion de la santé remet en cause, le terme a néanmoins pris vie de ses propres ailes, en se concentrant sur des évènements ou moments charnières qui permettent de reconfigurer les relations de pouvoir et les priorités des agendas. Dans une série récente de blogues, le BMJ a identifié 19 disrupteurs de la santé mondiale (7). Il s’agit notamment des épidémies dévastatrices (sida, syndrome respiratoire aigu sévère (SRAS), Ebola, maladies non transmissibles (MNT)), de très grands événements géopolitiques (la fin de la guerre froide, la Convention-cadre pour la lutte antitabac et l’Initiative Belt and Road), de changements de grande ampleur (l’urbanisation, les migrations, le changement climatique) et de nouveaux acteurs et phénomènes (le complexe médico-industriel et l’influence des grands donateurs privés/ONG).

Aujourd’hui, d’autres sujets sont considérés comme des disrupteurs : le néolibéralisme, les *Fridays for Future*, la COVID-19, l’initiative « *Wet’suwet’en Strong* », les *Marches pour la justice* et le mouvement des *Black Lives Matter*. On porte une attention renouvelée, à l’échelle mondiale, à l’équité (en santé) et aux chemins empruntés par ses détracteurs, à commencer par le colonialisme et le racisme. La promotion de la santé malheureusement continue d’être en grande partie politiquement et écologiquement aveugle (prétendant être « sans parti pris »), axée presque entièrement sur l’individu ou les relations interpersonnelles plutôt que sur les déterminants écologiques de la santé. La promotion de la santé a aussi des difficultés à s’attaquer de façon significative aux iniquités qui persistent dans nos sociétés. Même si le Rapport de l’Organisation mondiale de la Santé sur les Déterminants de la Santé (8) a ouvert la voie à une focalisation sur les inégalités de pouvoir afin de surmonter les inégalités sociales dans le domaine de la santé, nous avons besoin de nouvelles façons plus efficaces d’aborder ces problèmes par la recherche, la pratique et les politiques.

Nous constatons que les « déterminants sociaux » commencent à suivre la voie des « soins de santé primaires d’Alma Ata ». En fait Mills (9) a prédit les tendances dont nous avons été témoins au cours des dernières décennies : plutôt que d’engager politiquement les ressources communautaires pour améliorer la santé (ce qui était l’intention même de la Déclaration), une focalisation technocratique et médico-clinique de la théorie et de la pratique des soins de santé primaires semblent les avoir détournés de la population. De la même façon, les approches axées sur les déterminants sociaux sont en train de devenir des exercices dominés par la technocratie qui mettent l’accent sur les mesures, statistiques et indicateurs et les responsabilités économiques, alors que l’objectif principal du programme était – et demeure – sociopolitique. Il semble en être de même en ce qui concerne le potentiel émancipateur des Objectifs de développement durable (ODD). Ces bouleversements pourraient aussi contribuer à enraciner les systèmes actuels, comme on l’a vu dans le cas de l’alimentation avant la COVID-19 : des changements majeurs dans l’alimentation avaient renforcé la production industrielle et le commerce, plutôt que de mener à la souveraineté alimentaire (10). Il est donc encore plus important que prévu de s’organiser contre un tel enracinement de systèmes qui façonnent de manière négative la santé humaine. En reconnaissant les disrupteurs évoqués ci-dessus, on a posé un verre grossissant sur les relations entre ces différents événements et problématiques : les liens existants entre la crise climatique, les droits des peuples autochtones, la concentration de la richesse et la violence racialisée. Les événements eux-mêmes sont des disrupteurs, mais nous pourrions ne pas reconnaître que les liens entre eux peuvent être encore plus choquants et exiger des politiques qui recoupent (ou relient) les disrupteurs.

Ce pourrait être une vague parfaite pour les promoteurs de la santé (amoureux de surf). Cela nous permet de relier les points entre toutes ces questions et de braquer les projecteurs sur la santé et le bien-être dans toutes les politiques. Les disrupteurs identifiés dans l’article du BMJ ont façonné et façonneront ce que fait la gouvernance mondiale de la santé (depuis les épidémies jusqu’aux réfugiés climatiques), comment (depuis les campagnes de vaccination jusqu’aux accords commerciaux) et avec qui (depuis les acteurs étatiques traditionnels jusqu’aux fondations privées et aux mouvements sociaux). Pourtant, le changement et la gouvernance à l’échelle mondiale ont des dimensions locales et communautaires – et l’engagement entre les niveaux et les territoires est essentiel pour l’identification des changements dans les systèmes (c.-à-d. les politiques et les institutions). Par exemple, les villes (devraient) avoir comme objectif de repenser leur environnement bâti afin d’améliorer la qualité de l’air, la possibilité de marcher, le logement, le confort thermique et la sociabilité pour tous, et en particulier pour ceux qui subissent dans leur vie les conséquences de l’accumulation des inégalités. Les États doivent chercher des moyens d’améliorer l’accès aux soins de santé et aux services sociaux pour les plus démunis. La santé et le bien-être sont la colle qui aide à relier les points entre les disrupteurs car ils se traduisent tous par des résultats de santé aggravés et des inégalités de santé accrues. Pour les promoteurs de la santé, cela signifie renforcer la santé, le bien-être et l’équité dans d’autres politiques, en s’engageant avec des acteurs qui ont d’autres problèmes au cœur de leurs préoccupations, comme les parties prenantes engagées dans les questions environnementales, les urbanistes, les militants dans le domaine social, les industries de l’infrastructure, etc. La conférence offrira de nombreuses opportunités d’apprendre comment des promoteurs de la santé ont travaillé avec des professionnels dévoués provenant de différents domaines de politiques publiques. En fait, la conférence pourrait bien montrer que la promotion de la santé peut tout à fait vivre en dehors du secteur de la santé.

## Innover en brisant les silos entre nos pratiques, systèmes, recherches et politiques

Le deuxième sous-thème offre d’autres façons de penser et de travailler en promotion de la santé, et fait suite à la *Déclaration de 2019 des peuples autochtones de Waiora en faveur de la santé planétaire et du développement durable*. Cette déclaration, adoptée lors de notre dernière conférence mondiale, a appelé les communautés de la promotion de la santé, à l’échelle mondiale, à s’ouvrir aux peuples autochtones, et à privilégier leurs voix et leurs connaissances en prenant des mesures pour apaiser notre relation avec tous les êtres de la Terre Mère et privilégier le développement durable. Des siècles d’expansion impérialiste ont créé des systèmes et des institutions qui façonnent l’injustice économique, sociale et sanitaire généralisée, systémique et continue. Les peuples autochtones à travers le monde, en particulier, en souffrent de façon disproportionnée – leur culture, leurs liens familiaux, leur développement durable, leur écologie et leurs systèmes de connaissances ont été délibérément et clandestinement détruits. Les inégalités en santé sont donc le fruit d’une oppression systématique à long terme des peuples autochtones et de leurs modes de savoir (y compris de leurs façons de promouvoir la santé). Toutefois, la décolonisation de la promotion de la santé va au-delà d’une attention particulière portée aux peuples autochtones. Il faut créer des espaces pour différentes traditions épistémologiques qui déterminent la façon dont nous voyons le monde, la façon dont nous nous organisons dans celui-ci, les questions que nous posons et les solutions que nous recherchons. À mesure que nous intégrons d’autres épistémologies, nous reconnaissons l’importance de collaborer véritablement avec ceux qui ont souvent été « les étudiés » pour prendre part à la recherche dans l’intérêt de tous en utilisant des approches de recherche participatives et communautaires. De telles approches participatives nous obligent à réfléchir à notre positionnement dans la recherche et à réfléchir aux façons dont nous pouvons mieux faire entendre la voix, les besoins et les priorités des communautés en tant qu’alliés (11). *Waiora* nous aide également à comprendre certains des problèmes liés à l’idéologie néolibérale actuelle et, plus largement, à notre idéologie et à notre système capitalistes qui privilégient l’extraction des ressources et l’accumulation individuelle de richesses, plutôt que les responsabilités et la réciprocité.

Erondu *et al*. (12) ont récemment affirmé, alors qu’ils se penchaient sur une institution prestigieuse de santé publique, que « l’héritage colonial et le néo-colonialisme — définis par certains universitaires comme le renforcement des pratiques colonialistes de contrôle et d’influence par des actions, comportements, attitudes et croyances, la plupart du temps inconscientes — sont les fondements d’un modèle opérationnel systémique qui façonne les possibilités de carrière, les partenariats de recherche et les pratiques d’enseignement ». Ce regard colonial – ou « étranger » (13) – est omniprésent et ne constitue pas seulement un héritage durable de l’ambition impérialiste de quelques puissances blanches du Nord. Elle est plus insidieuse que cela et s’étend à la domination d’un système de connaissance particulier – cartésien. Mweemba *et al*. (14) montrent comment la sous-représentation systémique et systématique du Sud mondial maintient une illusion de supériorité coloniale – même si les « colonies » en tant qu’entités appartiennent pour la plupart au passé. La décolonisation n’est donc pas simplement la reconnaissance et l’excuse d’un paradigme capitaliste blanc. Il s’agit aussi de décentrer la blancheur, d’utiliser des outils d’équité raciale et de porter le discours de la décolonisation aux pays et aux populations du Sud.

Pour décoloniser la promotion de la santé et élaborer des politiques et des programmes de santé plus efficaces et culturellement adaptés, il faut que ce soit les communautés qui dirigent effectivement le processus d’élaboration des politiques. Il faut que leur engagement et leur participation soient réellement assurés. Nous devons remettre en question l’idée que les résultats de la recherche des sociétés occidentales dominantes (le Nord mondial) sont directement applicables dans d’autres contextes. Au contraire, nous devons générer des connaissances au sein même des communautés autochtones et minoritaires, avec elles et pour elles, afin de promouvoir l’équité en santé. La recherche impliquant des chercheurs autochtones et des membres de la communauté est nécessaire pour combler et refermer le fossé (15). De tels processus de décolonisation de la recherche montrent la voie à suivre pour co-créer de l’intelligence et changer la dynamique de pouvoir pour soutenir des innovations profondes et des changements radicaux. Les outils de la promotion de la santé devraient s’approprier les innovations propres aux méthodes de recherche autochtones comme la narration, le Dadirri et le modèle dit du « double regard » (16).

## Innovation émancipatrice

Dans la perspective de l’ « IUHPE2022 », il faut que la communauté mondiale de la promotion de la santé (y compris l’UIPES et d’autres institutions, mais aussi les décideurs politiques, les activistes et des institutions essentielles) identifie les principales innovations clés susceptibles de changer les façons de considérer les problèmes et leurs solutions. Nous devons commencer à identifier les personnes, les communautés et leurs réseaux qui peuvent conduire le changement – au niveau des politiques et des systèmes. L’innovation commence souvent à petite échelle et prend du temps à se répandre. C’est le réseautage qui va lui permettre d’être découverte, reconnue et diffusée. Il faut que l’ « IUHPE2022 » permette cela.

D’anciens concepts et technologies (comme l’intelligence artificielle) ont besoin d’être rafraîchis avec une optique forte de promotion de la santé, de bien-être et d’équité (ce pourrait être l’adoption de la Déclaration de Montréal pour un développement responsable de l’intelligence artificielle (17) à l’ « IUHPE2022 »). De la même façon, la mobilisation des mouvements sociaux en faveur de l’équité et du bien-être fait déjà partie de notre répertoire. La question de savoir si nous le faisons toujours bien et de manière responsable, mérite un examen critique. Le réseautage et l’engagement à l’échelle mondiale (à travers les médias sociaux) créent de nouvelles occasions de faire entendre plus de voix, sinon toutes. Le leadership inspiré et l’apprentissage par la pratique (18) doivent faire partie intégrante du changement de politique.

Un autre domaine d’innovation en promotion de la santé consiste à recadrer de manière plus significative et plus délibérée les systèmes de pouvoir et les intérêts qui favorisent le maintien des façons de travailler, de faire et d’arranger les « qui obtient quoi, pourquoi et quand » du jeu politique. Ces questions sont au cœur même de la promotion de la santé et pourtant, mis à part certains idéologues persévérants en marge de notre mouvement, nous n’avons pas réussi à intégrer de nouvelles idées comme l’éconologie (19), la consucratie (20), les changements intergénérationnels transformateurs et la polarisation des systèmes de valeurs dans un plan d’action fort.

Nous innovons de différentes manières et nous vous invitons à vous retrouver sur le Territoire traditionnel des Haudenosaunee/Anishinaabe de Tiohtià:ke (Montréal) en mai 2022. Le bouleversement de la syndémie a créé l’opportunité d’organiser une conférence différente, en mode hybride (en personne et de manière virtuelle) qui permet à beaucoup plus de voix d’être entendues et à plus d’esprits d’être réunis. Il faut que tous nous nous aidions à poursuivre des changements fructueux, à décoloniser le patrimoine commun de l’humanité et à innover pour améliorer la santé, le bien-être et l’équité. Nous invitons les promoteurs de la santé, les communautés, les activistes, les universitaires et, plus important encore, les opérateurs des politiques publiques à contribuer à transformer notre monde au profit de toutes les Nations et de toutes nos relations avec la Terre Mère.
